# Late Presentation of Recurrent Monomorphic Ventricular Tachycardia following Minimally Invasive Mitral Valve Repair due to Epicardial Injury

**DOI:** 10.1155/2014/976494

**Published:** 2014-01-06

**Authors:** Harry L. South, Moses Osoro, Tjuan Overly

**Affiliations:** ^1^Department of Medicine, Division of Cardiology, Graduate School of Medicine, University of Tennessee, Knoxville, TN 37920, USA; ^2^Department of Medicine, Division of Internal Medicine, Graduate School of Medicine, University of Tennessee, Knoxville, TN 37920, USA; ^3^Heart Lung Vascular Institute, University of Tennessee Medical Center, 1940 Alcoa Highway, Suite E 310, Knoxville, TN 37920, USA

## Abstract

We report a 73-year-old male with late onset monomorphic ventricular tachycardia following mitral valve repair (MVR). Typically, injury to epicardial arteries following mitral valve repair/replacement presents immediately as ventricular tachycardia/fibrillation, difficulty weaning from cardiopulmonary bypass, worsening ECG changes, increasing cardiac biomarkers, or new wall motion abnormalities. Our case illustrates a “late complication” of a distorted circumflex artery following mitral valve repair and the importance of early diagnostic angiography and percutaneous intervention.

## 1. Introduction

In experienced centers mitral valve repair has low mortality rates and excellent outcomes. The Cleveland Clinic and Mayo Clinic report 30 day mortality rates of 0.3% and 0.9%, respectively [1, 2]. Injury to the circumflex artery during MVR is a recognized complication, albeit rare. The proximity of the circumflex artery to the anterolateral commissure of the mitral valve predisposes the artery to injury during mitral valve surgery [3, 4]. Acute occlusion of the circumflex artery may manifest immediately as ventricular arrhythmia, difficulty weaning from cardiopulmonary bypass, ST elevation myocardial infarction, or new wall motion abnormality [5]. We report a late onset sustained monomorphic ventricular tachycardia (SMVT) secondary to distortion of the circumflex artery following mitral valve repair.

## 2. Case Report

A 73-year-old male with mitral regurgitation presented to his primary cardiologist with symptoms of increasing dyspnea on exertion. As part of the diagnostic workup, a transthoracic echocardiogram (TTE) was performed which revealed significant pulmonary hypertension (estimated pulmonary artery pressures of 50–55 mmHg), not previously seen on prior TTE. Moderate mitral valve insufficiency with prolapse of the posterior leaflet and normal right ventricular function was also present.

Past medical history was significant for hyperlipidemia, chronic obstructive pulmonary disease, chronic kidney disease (stage 3), and benign prostatic hypertrophy.

The decision was made by the primary cardiologist to refer the patient for mitral valve repair. In anticipation of the aforementioned procedure a left and right cardiac catheterization was performed which showed a left dominant system with nonobstructive coronary artery disease, normal left ventricular function, right ventricular pressures of 68/9 mmHg, pulmonary artery pressures of 63/30 mmHg, and a pulmonary capillary wedge pressure of 21 mmHg. The patient was then referred for cardiothoracic surgeon consultation.

After evaluation by the cardiothoracic surgeon, the patient underwent robotically assisted mitral valve repair. The extended transseptal (ETS) approach was used for annuloplasty ring insertion and triangular resection of the flail P2 segment of the mitral valve. Epicardial pacing leads were placed on the right ventricle and epicardial pacing initiated after the ring was sutured in place. The patient tolerated the procedure well without any perceived complications at that time.

On postoperative day 0, the patient remained intubated and required intermittent epicardial pacing. A postoperative electrocardiogram (ECG) showed sinus rhythm, first degree atrioventricular block (PR interval = 320 msec), and 3.5–4 mm ST elevations in leads II, II, and aVL with reciprocal changes in the anterolateral leads. Serial ECG showed resolution of ST elevation; the patient was hemodynamically stable and was successfully weaned off ventilator support; therefore, the decision was made to manage the patient medically. On postoperative day 4, cardiology consultation was obtained because the patient required continuous epicardial pacing for underlying third degree atrioventricular block. At the time of the interview the patient was alert, asymptomatic, and hemodynamically stable without ST elevation/depression on ECG. Therefore, the patient was observed for 48 hours and the decision was made to proceed with pacemaker insertion.

After evaluation by an electrophysiologist, a dual chamber permanent pacemaker (PPM) was placed on postoperative day 6. The patient also developed atrial fibrillation which was treated with successful direct current cardioversion and initiation of oral amiodarone therapy.

In the evening after placement of the PPM, the patient began having intermittent nonsustained ventricular tachycardia. The pacemaker was interrogated and found to be in normal operating condition. The leads were scrutinized under fluoroscopy and appeared to be without fracture and in proper anatomical position. The following day, postoperative day 7, the patient had recurrent episodes of SMVT with ventricular rates in the range of 150–160 bpm. Therefore, intravenous amiodarone therapy was started and the pacemaker was upgraded to a pacemaker/implantable cardioverter-defibrillator (PPM/ICD) on postoperative day 8. Despite the aforementioned treatment, on postoperative day 10, the patient continued to have SMVT with spontaneous termination. The PPM/ICD was interrogated to determine if the etiology of the ventricular tachycardia was pacemaker-mediated; however, the PPM/ICD was functioning properly.

**Figure 1:**
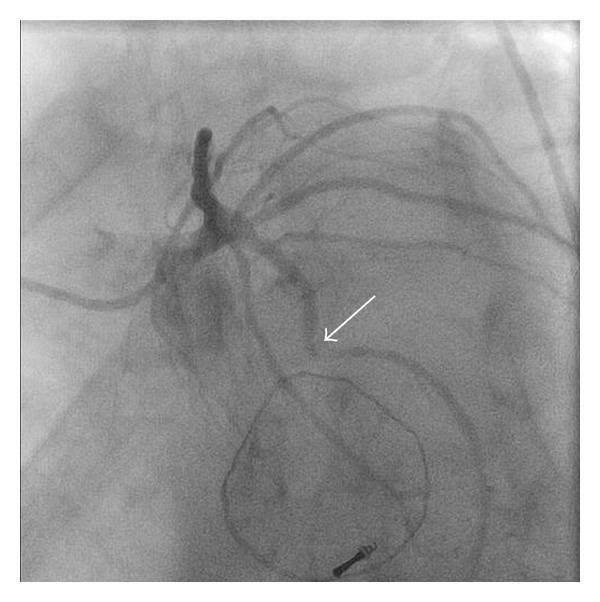
Kinked circumflex artery (arrow) secondary to suture distortion from a recently repaired mitral valve. Left anterior oblique with caudal angulation.

**Figure 2:**
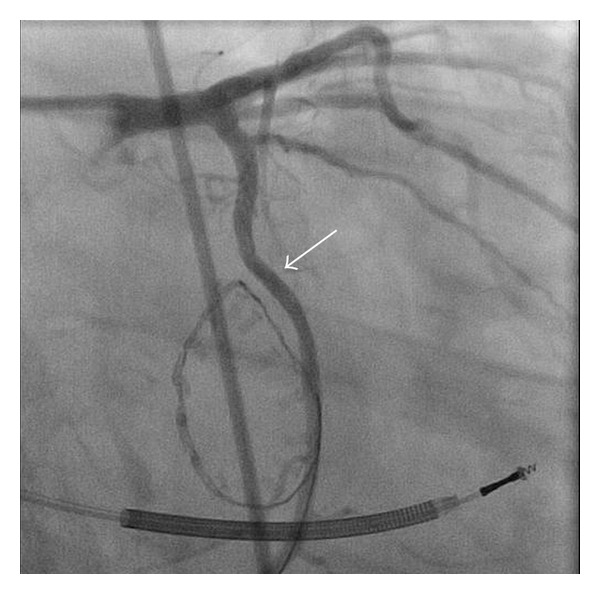
Final results of percutaneous coronary intervention with bare metal stent (arrow) of the previously distorted circumflex artery. Right anterior oblique with caudal angulation.

The patient was taken to the cardiac catheterization lab on postoperative day 11 for left cardiac catheterization which revealed tenting of the mid circumflex artery, with 99% stenosis and TIMI grade I flow distally (Figure 1) and normal left ventricular ejection fraction. A 2.25 mm bare metal stent was placed within the mid circumflex with excellent angiographic results and TIMI grade III flow distally after the intervention (Figure 2). The patient was started on dual antiplatelet therapy and was discharged in sinus rhythm and stable condition on postoperative day 12. The patient was in stable condition during a 4 month followup and interrogation of the ICD revealed one episode of ventricular tachycardia that was successfully treated (shock therapy).

## 3. Discussion

Injury to epicardial coronary arteries following atrioventricular valve repair is a rare complication. Owing to the proximity of the circumflex artery to the mitral valve, the left dominant systems are especially prone to injury following valve repair or replacement [3, 5, 6]. Patel et al. recently reported injury to both the circumflex and right coronary arteries in a patient who underwent concomitant mitral and tricuspid valve repair. However, the rates of such complications are low. In a study of 222 patients undergoing mitral valve repair, 4 patients (1.8%) had postoperative ischemia secondary to suspected injury to the circumflex artery, although only 1 of the 4 patients (0.4%) required revascularization [2]. Prompt diagnosis is essential as myocardial infarction with subsequent left ventricular ruptured and death has been described following kinking of the circumflex artery with mitral valve replacement [7].

Proposed mechanisms of injury to the epicardial coronary vessel following mitral valve surgery include air embolism, kinking of the artery from traction of the annuloplasty ring suture, coronary spasm, and percutaneous coronary intervention resulting in circumflex to left atrial fistula formation [5, 8]. Onset of symptoms is typically reported immediately (intraoperative to a few hours postoperative). Presentations vary from immediate ventricular tachycardia/fibrillation, difficulty weaning from cardiopulmonary bypass, worsening ECG changes and increasing cardiac biomarkers, and new wall motion abnormalities. Our case is unique in that it initially presented as transient ECG changes, managed conservatively with medical management, but later presented as late-onset SMVT.

Initially, our patient had 3.5–4 mm ST elevation in leads II, III, and aVL in the “immediate” postoperative period. However, the decision was made to manage the patient medically given early resolution of ECG changes, lack of clinical symptoms of angina or heart failure, and hemodynamic stability. When ECG changes were initial noted during the early postoperative period an echocardiogram to look for wall motion abnormalities and cardiac biomarkers may have been helpful to determine if immediate revascularization was indicated. The myocardial infarction presumably resulted in third degree atrioventricular block requiring pacemaker insertion.

The importance of developing a broad differential is paramount as cardiac catheterization prior to pacemaker upgrade to a PPM/ICD may have been beneficial. Prior case reports show angiographic findings of a total occluded circumflex and kinked right coronary artery following concomitant repair of the mitral and tricuspid valve. Initially, the patient was managed by percutaneous intervention to the circumflex artery alone; however, 5 days later the patient continued to have recurrent ventricular arrhythmias, prompting repair of the kinked right coronary artery. Similarly, our patient continued to have recurrent monomorphic ventricular tachycardia secondary to distortion of the circumflex artery and poor distal flow. An alternative etiology could be formation of a scar secondary to myocardial infarction with peri-infarct ischemia due to the distorted circumflex artery.

## 4. Conclusion

Albeit rare, early recognition of epicardial coronary artery vessel injury is paramount to avoid potentially fatal complications and morbidity following mitral valve repair [8]. We agree with previous authors and recommend a low threshold to perform coronary angiography in patients presenting after mitral valve repair (or replacement) with ST elevation, new wall motion abnormalities, ventricular arrhythmias, high degree atrioventricular block, or hemodynamic instability. Our case demonstrates that recurrent ventricular arrhythmias can occur as a “late sequel” of kinked epicardial arteries and the importance of early revascularization following epicardial injury during mitral valve repair.
